# High‐sensitivity CRP is elevated in pregnant women with overweight and obesity and modulated by gestational weight gain

**DOI:** 10.1111/aogs.15135

**Published:** 2025-05-07

**Authors:** Ann‐Christin Tallarek, Tanja Zeller, Alina Goßling, Caroline Kellner, Gudula Hansen, Mirja Pagenkemper, Charlotte Humke, Evelyn Annegret Huhn, Christopher Urbschat, Petra Clara Arck, Anke Diemert

**Affiliations:** ^1^ Department of Obstetrics and Fetal Medicine University Medical Centre Hamburg‐Eppendorf Hamburg Germany; ^2^ Junior Research Center for Reproduction: Sexual and Reproductive Health in Overweight and Obesity (SRHOO) University Medical Center Hamburg‐Eppendorf Hamburg Germany; ^3^ University Center of Cardiovascular Science, University Heart and Vascular Center Hamburg Germany; ^4^ Department of Cardiology University Heart and Vascular Center Hamburg Germany; ^5^ Division for Experimental Feto‐Maternal Medicine University Medical Centre Hamburg‐Eppendorf Hamburg Germany; ^6^ Hamburg Center for Translational Immunology Hamburg Germany

**Keywords:** C‐reactive protein, gestational weight gain, low‐grade inflammation, obesity, overweight

## Abstract

**Introduction:**

The global obesity epidemic presents a growing challenge in perinatal medicine and obstetrics, as it is associated with a higher risk of adverse maternal and perinatal outcomes. In addition to metabolic disturbances, obesity contributes to chronic low‐grade inflammation. This study aims to investigate the relationship between maternal overweight, obesity, and excessive weight gain during pregnancy and high‐sensitivity C‐reactive protein (hs‐CRP), an acute‐phase reactant of inflammation and infection in maternal serum.

**Material and Methods:**

The PRINCE (Prenatal Identification of Children's Health) study is a prospective longitudinal pregnancy cohort conducted at the University Medical Centre Hamburg‐Eppendorf in Germany. In this study, biosamples and metadata were collected from 2011 to 2023. Hs‐CRP levels were measured in each trimester among 582 healthy, low‐risk women. Participants were categorized into weight classes based on their body mass index. Weight gain during pregnancy was evaluated according to the recommendations of the Institute of Medicine. For statistical analysis, the Wilcoxon and Kruskal–Wallis tests were employed for continuous variables, whereas the Mann–Whitney test was used for binary groups.

**Results:**

Hs‐CRP levels are significantly higher in overweight and obese pregnant women throughout their pregnancy. During the first trimester, hs‐CRP compared to those of normal‐weight individuals (mean 3.4 mg/L; 95% CI 3.1 to 3.7) showed a 1.7‐fold increase in overweight (mean 5.9 mg/L; 95% CI 5.0 to 6.7; *p* < 0.0001) and a 2.7‐fold increase in obesity (mean 9.3 mg/L; 95% CI 7.6 to 11.0; *p* < 0.0001). Excessive weight gain during pregnancy is more common among overweight women (67%) and is associated with 1.5‐fold heightened hs‐CRP levels in the third trimester (mean 5.6 mg/L; 95% CI 4.7 to 6.4), compared to overweight women who follow the recommended weight gain guidelines (mean 3.7 mg/L; 95% CI 2.9 to 4.4; *p* = 0.009).

**Conclusions:**

Overweight and obesity, along with excessive gestational weight gain in individuals with preexisting overweight status, correlate with elevated levels of hs‐CRP during pregnancy. This phenomenon indicates chronic low‐grade inflammation in adipose tissue.

## Graphical abstract

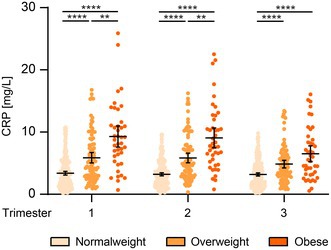

**Abbreviations**
BMI: body mass index
Hs‐CRP: high‐sensitivity C‐reactive protein
PRINCE: Prenatal Identification of Children's Health

**Key message**

The adverse inflammatory phenotype in overweight and obese pregnant women is characterized by markedly elevated levels of hs‐CRP throughout pregnancy and partially modified by maternal gestational weight gain. This highlights the interplay between obesity, inflammation, and weight management during pregnancy.

## 1 INTRODUCTION

The global obesity epidemic observed in recent years has significant implications for reproductive outcomes.^1^, ^2^ Numerous studies have shown that both prepregnancy obesity and excessive gestational weight gain adversely affect feto‐maternal health during pregnancy and after childbirth.^3^, ^4^, ^5^, ^6^, ^7^ This situation places a considerable burden on affected individuals and the healthcare system, highlighting the urgent need for intensified research to address knowledge gaps regarding causal relationships in pregnancy.

In nonpregnant individuals, C‐reactive protein (CRP) has consistently been associated with overweight and obesity in various epidemiological studies.^8^, ^9^ This association can be attributed to the activation of inflammatory pathways within adipose tissue, resulting in chronic low‐grade inflammation.^10^

CRP, an acute‐phase reactant associated with inflammation and infection, is widely used as a clinical biomarker for inflammation. It plays a significant role in various low‐grade inflammatory processes, particularly in the pathogenesis of cardiovascular diseases.^11^ Notably, Ridker et al. recently demonstrated that elevated levels of high‐sensitivity (hs)‐CRP in healthy women can predict future cardiovascular events.^12^

In the context of pregnancy, low‐grade inflammation has been linked to a variety of pregnancy complications, including preterm birth, preeclampsia, and gestational diabetes. It is also associated with increased long‐term health risks for both mothers and their offspring.^13^ While CRP has been investigated concerning severe pregnancy complications such as premature rupture of membranes and chorioamnionitis,^14^ there is still limited understanding of low‐grade, subclinical inflammatory processes and related surges of CRP.

The objective of the present prospective cohort study was to examine how maternal body mass index (BMI) and gestational weight gain influence the trajectory of systemic hs‐CRP levels throughout pregnancy. This research aims to enhance the understanding of low‐grade chronic inflammation in pregnancies affected by overweight and obesity.

## 2 MATERIAL AND METHODS

### 2.1 Study design and recruitment

Biosamples and metadata from the PRINCE study (Prenatal Identification of Children's Health) have been utilized for the current analyses. The PRINCE study is a prospective longitudinal pregnancy study that commenced in 2011 at the University Medical Centre Hamburg‐Eppendorf, Germany. Several studies with results from the cohort have been published previously, primarily addressing immunological and psychological factors and their impact on fetal programming.^15^, ^16^ Inclusion criteria were nonsmoking women aged 18 or older with a viable singleton pregnancy of gestational week (gw) 12 + 0 to 14 + 6; exclusion criteria comprised chronic infections (HIV, hepatitis B/C) or serious diseases of the immune system, known drug or alcohol abuse, fetal anomalies, multiple pregnancies, or pregnancies induced by assisted reproductive technology. At 12–14, 22–24, and 34–36 weeks of gestation, materno‐fetal ultrasound was conducted, a maternal venous blood sample was taken, and pregnancy progression, health status, and anthropometric data of the mother were documented. Anthropometric indices of the neonate were also obtained, and cord blood was collected. Pregnancy complications and outcomes were recorded using a self‐reported questionnaire and evaluated from the electronic patient record.

The maternal height and weight were measured to the nearest 0.5 cm and 0.1 kg, respectively, using the same method. The BMI ranges were applied following WHO recommendations.^17^ Pregnancy weight gain was assessed according to the guidelines from the US Institute of Medicine: 12.5–18 kg for underweight women, 11.5–16 kg for normal‐weight women, 7–11.5 kg for overweight women, and 5–9 kg for obese women.^18^

### 2.2 Biological sampling

At every visit during pregnancy, a venous blood sample was taken from the mother via peripheral venipuncture, and cord blood was collected at birth following cord clamping. Serum samples were stored at −80°C until needed and were maintained at 4°C after thawing.

### 2.3 Analysis of CRP in serum

Commercially available MULTIGENT CRP Vario Assay kits (CRPVa) were employed to assess CRP levels using the high‐sensitive method (CRP16) in maternal serum. The assays were conducted on the Abbott Architect c8000. A CRP value of ≤5 mg/L is regarded as the recommended range. The measurement range for the high‐sensitive method was between 0.1 and 160 mg/L. The intercoefficient variation was calculated at three different levels (low/medium/high). Intercoefficient variation % values were 1.24 (at low levels using samples with concentrations of 2.59–3.89 mg/L), 0.92 (at medium levels using samples with concentrations of 6.76–10.10 mg/L), and 1.6 (at high levels using samples with concentrations of 22.2–33.3 mg/L). The precision of the assay is indicated by an intracoefficient variation of 0.82. This intracoefficient variation was measured using a lab‐internal serum sample with unknown concentration and was assessed 15 times.

### 2.4 Statistical analyses

**FIGURE 1:**
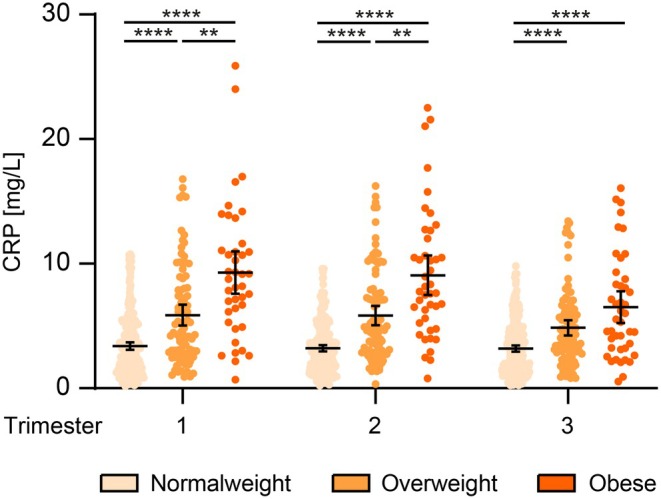
Hs‐CRP in the individual trimesters depending on maternal BMI before pregnancy. Normal weight: BMI 18.5–24.9; overweight: BMI 25–29.9; obese: BMI >30. Scatterplot showing mean (thick horizontal line) with 95% confidence interval (CI). **p < 0.01, ****p < 0.0001.

For the baseline characteristics and metabolic parameters of the study population, all statistical analyses were performed using R version 3.0.4 (R Foundation for Statistical Computing). Between‐group comparisons for two groups regarding continuous variables were conducted using the Mann–Whitney test. For more than two groups, unadjusted between‐group comparisons of continuous variables were carried out with the Kruskal–Wallis test. As this method is based on ranks, it is robust against outliers. Statistical comparisons between binary groups were performed using the chi‐squared test. Continuous variables are shown as medians (25th percentile, 75th percentile), whereas binary variables are displayed as median counts (frequencies). Statistical analysis of the data for the figures and the creation of the figures were conducted using Prism 9.5.1 (GraphPad, La Jolla, CA, USA). For Figure 1 and Figure S1, statistical significance was calculated using the Kruskal–Wallis test with Dunn's correction. For Figure 3, statistical significance was calculated using the Mann–Whitney test. Outlier analysis for all figures was performed using the ROUT method, *Q* = 1%. All figures show scatter plots with mean and 95% confidence intervals. A *p*‐value of <0.05 was considered significant.

## 3 RESULTS

### 3.1 Clinical characteristics

The study population comprised 582 women in a low‐risk cohort. In total, 48% of the women enrolled in the study (*n* = 280) were nulliparous. The median age was 32 years (25th–75th percentile 30–35 years).

According to the WHO criteria for BMI,^17^ the mean BMI of our study population was within the normal range (BMI 23.5; 21.5–25.9). More specifically, 2% of women (*n* = 10) were underweight (BMI < 18.5), approximately two‐thirds (66%) of the study population (*n* = 381) were of normal weight (BMI 18.5–24.9), 24% (*n* = 138) were overweight (BMI 25–29.9), and 9% (*n* = 52) had a BMI ≥30, thus categorizing them as obese. Furthermore, 36% of the PRINCE study participants (*n* = 204) demonstrated a gestational weight gain below the reference range suggested by the Institute of Medicine,^18^ whereas 36% (*n* = 203) gained weight within this range. Excessive weight gain was observed in 28% (*n* = 160) of the participants.

The baseline characteristics, including the pregnancy outcomes of the study population, are presented in Table 1.

**TABLE 1:** Baseline characteristics of the study population.

	All	Underweight	Normal weight	Overweight	Obese	*p*‐Value
	*n* = 582	*n* = 10 (2%)	*n* = 381 (66%)	*n* = 138 (24%)	*n* = 52 (9%)	
Sociodemographic						
Maternal age – median years (IQ)	32 (30–35)	33 (30–35)	32 (30–35)	32 (30–35)	3337 (30–35)	0.98
Nulliparous	280 (48%)	4 (40%)	186 (49%)	68 (49%)	21 (40%)	0.67
BMI median (IQ)	23.5 (21.5–25.9)	18.3 (17.6–18.4)	22.3 (20.9–23.6)	26.4 (25.7–27.7)	33.8 (31.4–36.2)	<0.0001
Weight gain total—median, kg (IQ)	11.5 (9.4–14.2)	13.5 (11–16.2)	11.2 (9.2–13.7)	12.7 (10.6–15)	10.3 (6.6–12.3)	<0.0001
Below IOM recommendation	204 (36%)	5 (50%)	188 (51%)	4 (3%)	7 (14%)	<0,0001
Within IOM recommendation	203 (36%)	5 (50%)	143 (39%)	41 (30%)	14 (27%)	0.14
Above IOM recommendation	160 (28%)		40 (11%)	90 (67%)	30 (59%)	<0.0001
Pregnancy outcome						
Gestational week—median (IQ)	39.0 (39.0–40.0)	39.0 (38.0–39.75)	39.0 (39.0–40.0)	39.0 (39.0–40.0)	39.0 (39.0–40.0)	0.20
Preterm delivery (gw <37 + 0)	18 (3%)	0	11 (3%)	5 (4%)	2 (4%)	0.74
Preeclampsia	6 (1%)	0	2 (1%)	2 (2%)	2 (4%)	0.046
Gestational diabetes	27 (5%)	0	10 (3%)	8 (6%)	9 (17%)	0.00015
Cesarean section	135 (24%)	1 (10%)	83 (22%)	39 (29%)	11 (22%)	0.33
Fetal sex: boy	293 (50%)	6 (60%)	190 (50%)	70 (51%)	26 (51%)	0.95
Birth weight—median g (IQ)	3540 (3180–3830)	3430 (3220–3660)	3470 (3140–3750)	3620 (3390–3910)	3590 (3230–3920)	0.00053
Head circumference—median g (IQ)	35 (34–36)	35 (34–35)	35 (34–36)	36 (35–36.5)	35 (34–36)	0.014

*Note*: Discrepancies in sample sizes are due to missing data. Continuous variables are shown as medians (25th percentile, 75th percentile) and compared using the Kruskal–Wallis test. Binary variables are shown as counts (frequencies) and compared using the Chi‐square test.

### 3.2 Metabolic parameters

Table S1 presents the results of the maternal metabolic biomarkers determined in the first trimester. The median values of the various measured biomarkers for systemic inflammation, lipid, and glucose metabolism were all in the recommended range, reflecting that the PRINCE cohort is a low‐risk group of pregnant women.

### 3.3 Overweight and obesity are associated with an adverse inflammatory phenotype

In pregnant women who are overweight or obese, we observed significantly higher CRP values compared to those of normal weight. Using the linear regression model presented in Table S2, we determined that a statistically significant association between maternal age and elevated CRP values could be excluded. The differences in CRP levels were most pronounced during the first and second trimester. However, values remained within the recommended range or were only slightly elevated, indicating that they were not pathologically elevated. In the first trimester, CRP was 1.7‐fold higher in overweight women (mean 5.9 mg/L; 95% CI 5.0 to 6.7; *p* < 0.0001) and 2.7‐fold higher in those with obesity (mean 9.3 mg/L; 95% CI 7.6 to 11.0; *p* < 0.001) compared to normal‐weight women (mean 3.4 mg/L; 95% CI 3.1 to 3.7). In the second trimester, the increases were 1.8‐fold (mean 5.8 mg/L; 95% CI 5.1 to 6.6; *p* < 0.0001) and 2.8‐fold (mean 9.1 mg/L; 95% CI 7.5 to 10.7; *p* < 0.0001) compared with normal‐weight (mean 3.2 mg/L; 95% CI 3.0 to 3.5). In the third trimester, the increase was 1.5‐fold (mean 4.9 mg/L; 95% CI 4.2 to 5.5; *p* < 0.0001) and 2‐fold (mean 6.5 mg/L; 95% CI 5.2 to 7.8; *p* > 0.00) in comparison to normal‐weight (mean 3.2 mg/L; 95% CI 2.9 to 3.4). Figure 1 shows the hs‐CRP in the individual trimesters depending on the mother's initial BMI.

### 3.4 Overweight and obese women gain disproportionate weight during pregnancy

**FIGURE 2:**
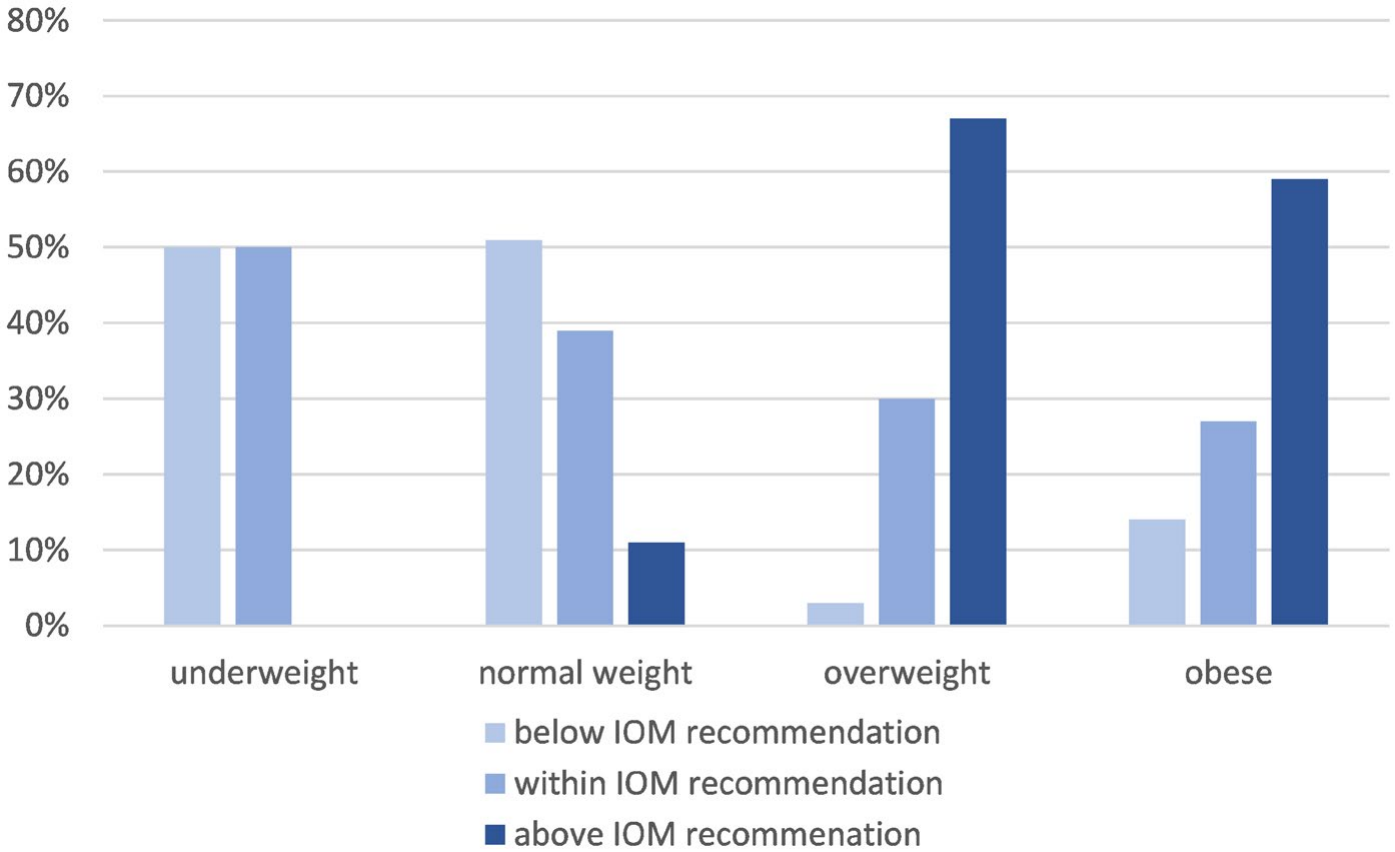
Weight gain during pregnancy depending on maternal BMI before pregnancy. Normal weight: BMI 18.5–24.9, overweight: BMI 25–29.9, obese: BMI >30. IOM Pregnancy Weight Guidelines: recommended weight gain in normal weight 11.5–16 kg, in overweight 7–11.5 kg, and in obese 5–9 kg.^18^

We observed that two‐thirds of the overweight women (67%; *n* = 90) in our population and more than half of the obese women (59%; *n* = 30) experienced excessive gestational weight gain according to Institute of Medicine guidelines, whereas only 11% of normal‐weight women (*n* = 40) exhibited excessive weight gain. The differing numbers of pregnant women with excessive weight gain across the individual BMI categories are statistically significant (*p* < 0.0001) and are illustrated in Figure 2.

### 3.5 Modification of CRP levels during pregnancy depends on the initial BMI and weight gain in pregnancy

**FIGURE 3:**
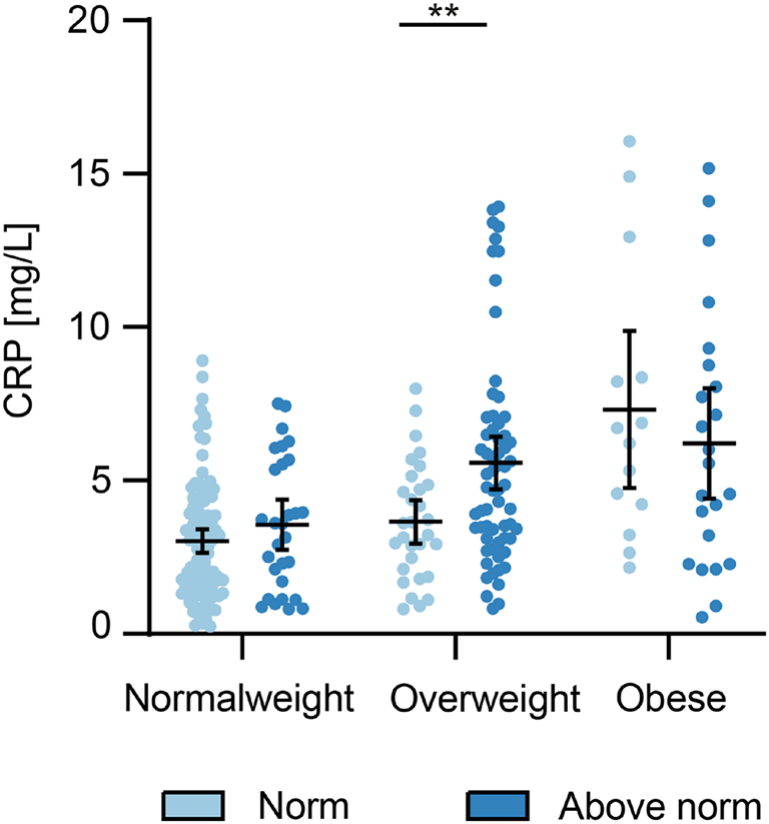
Hs‐CRP depending on weight gain in pregnancy in women in different BMI classes. Normal weight: BMI 18.5–24.9, overweight: BMI 25–29.9, and obese: BMI >30. Normal weight gain in normal weight 11.5–16 kg, in overweight 7–11.5 kg, and in obese 5–9 kg. Scatterplot showing mean (thick horizontal line) with 95% CI, **p < 0.01.

In women with a normal BMI and obese women , no association between elevated hs‐CRP levels in the third trimester and excessive weight gain could be demonstrated. However, in overweight pregnant women experiencing excessive weight gain , significantly elevated CRP levels were observed in the third trimester (Figure 3). Excessive weight gain during pregnancy led to a 1.5‐fold increase in hs‐CRP levels in the third trimester (mean 5.6 mg/L; 95% CI 4.7 to 6.4) compared to overweight women adhering to the recommended weight gain (n=31) (mean 3.7 mg/L; 95% CI 2.9 to 4.4; *p* = 0.009). Only a few overweight (*n* = 4) and obese (*n* = 7) women had gestational weight gain below the Institute of Medicine recommendations. Accordingly, no further statistical analyses were performed for these women.

### 3.6 Higher CRP levels do not correlate with increased birth weight

Overweight and obesity are related to GDM, and all are related to fetal macrosomia. However, no statistically significant associations between birth weight and CRP levels during any trimester were observed (Figure S1).

## 4 DISCUSSION

This study evaluated the relationship between overweight, obesity, gestational weight gain, and hs‐CRP in women during pregnancy.

Obesity is marked by metabolic dysfunction and low‐grade chronic inflammation.^19^, ^20^, ^21^ Consequently, it is associated with elevated levels of CRP, a marker of inflammation and cardiovascular disease.^22^ However, there are currently no studies that have systematically investigated the trajectory of CRP in overweight and obese pregnant women, nor the additional challenges posed by excessive gestational weight gain.

Our results show that, similar to nonpregnant individuals, overweight and obesity in pregnancy are associated with an unfavorable inflammatory phenotype compared to normal weight, mirrored by higher CRP levels in the first trimester. This finding is consistent with the role of adipose tissue in the production of interleukin‐6, an upstream progenitor of hs‐CRP.^23^ We demonstrated that the pro‐inflammatory state endures throughout pregnancy until delivery.

The inflammatory phenotype, which is considered adverse in nonpregnant individuals, is also present during pregnancy and is already evident in its early stages. This underscores the importance of focusing on preventive measures before pregnancy in this population. Research has demonstrated that a reduction in CRP can be achieved at least in nonpregnant individuals through both behavioral and medical methods, including increased physical activity, a Mediterranean diet, weight loss, smoking cessation, and pharmacological agents.^24^, ^25^

Low‐grade chronic inflammation during pregnancy raises concerns as a potential trigger for complications such as preterm birth and preeclampsia, while also posing risks to long‐term maternal health. Our study confirms that women with obesity have a significantly higher likelihood of developing preeclampsia. Published evidence by Louwen et al. indicates that deregulated metabolism and low‐grade inflammation in obese women severely impair placental function, suggesting a link between low‐grade inflammation in adipose tissue and the increased risk of preeclampsia associated with obesity.^26^ A systematic review and meta‐analysis conducted by Puttaiah and colleagues confirmed elevated levels of hs‐CRP in women affected by preeclampsia.^27^ Previous studies have established that statin medication administered before 20 weeks of gestation can prevent upregulation of inflammatory biomarkers and exhibit antioxidant effects in cases of preeclampsia.^28^, ^29^, ^30^ Given that preeclampsia is regarded as cardiovascular dysfunction, the recent study by Ridker and coworkers, which found that a single measurement of hs‐CRP in initially healthy women predicted cardiovascular events over a 30‐year period, warrants the attention of obstetricians.^12^

In our study, we found that a significant number of overweight and obese women experienced disproportionate weight gain during pregnancy. In 2009, the Institute of Medicine released guidelines for gestational weight gain that consider a woman's initial BMI.^18^ However, it appears that many women continue to gain weight beyond the recommendations.^31^ Current research suggests that excessive weight gain during pregnancy is associated with an increased risk of pregnancy complications for both the mother and the neonate.^32^, ^33^, ^34^, ^35^, ^36^

Our findings indicate that excessive weight gain during pregnancy in overweight women was associated with significantly elevated CRP levels in the third trimester compared to those with normal weight gain. In contrast, this effect was not observed in women of normal weight or those who were obese. It raises the question of whether the elevated CRP levels seen in obese pregnant women, which were already considerably beyond the specified range of the reference population, reflect a chronic inflammatory state that may be irreversible. Conversely, it is possible that the intact regulatory mechanisms in pregnant women of normal weight could effectively mitigate the inflammatory responses triggered by excessive weight gain.

The study presents strengths and limitations pertaining to its scope, data, and methods that merit discussion. Due to a substantial number of participants and the longitudinal design with high compliance, we could effectively document CRP levels and various influencing factors, such as BMI, throughout pregnancy. The PRINCE study was explicitly designed as a low‐risk cohort, which resulted in an underrepresentation of obese women. Additionally, severe pregnancy complications were infrequent, making it difficult to establish a definitive link between specific complications and low‐grade inflammation or CRP levels within our data set.

Future studies, especially translational studies, should investigate whether a causal relationship exists between low‐grade inflammation and adverse pregnancy outcomes in overweight or obese individuals. Establishing this connection could pave the way for utilizing inflammatory markers such as hs‐CRP as prognostic indicators in early pregnancy. Additionally, future initiatives aimed at managing weight gain during pregnancy should focus on both overweight and obese women.

Gynecologists, obstetricians, and general practitioners have a unique opportunity to initiate secondary preventive measures for women during pregnancy and their reproductive years, for example, controlled weight reduction measures. Such a proactive approach has the potential to significantly enhance long‐term cardiovascular health and immediate obstetric outcomes, making it an opportunity that should not be overlooked.

## 5 CONCLUSION

Our findings reveal for the first time that hs‐CRP, as an indicator of low‐grade chronic inflammation in overweight and obesity, is positively correlated with maternal BMI throughout pregnancy, with the most pronounced correlation in early pregnancy. Additionally, our results suggest that the inflammatory profile of overweight women may be particularly susceptible to excessive gestational weight gain. Future translational research should aim to determine whether there is a causal relationship between low‐severity inflammation and the risk of pregnancy complications in overweight and obese women.

## AUTHOR CONTRIBUTIONS

**Ann‐Christin Tallarek:** enrolled the subjects and collected samples and wrote the original draft. **Tanja Zeller:** coordinated sample collection, quality control, and assessments, was responsible for measuring biomarkers in all samples, reviewed and edited the manuscript, and secured funding. **Alina Goßling:** performed most of the analyses, processed the experimental data, and designed most of the figures, reviewed and edited the manuscript. **Caroline Kellner:** reviewed and edited the manuscript. **Gudula Hansen:** enrolled the subjects and collected samples, reviewed and edited the manuscript. **Mirja Pagenkemper:** enrolled the subjects and collected samples, reviewed and edited the manuscript. **Charlotte Humke:** edited the original draft. **Evelyn Annegret Huhn:** edited the original draft. **Christopher Urbschat:** performed most of the analyses, processed the experimental data, designed most of the figures, and reviewed and edited the manuscript. **Petra Clara Arck:** coordinated sample collection, quality control, and assessments and reviewed and edited the manuscript. **Anke Diemert:** developed the study and designed most of the experimental setups, enrolled the subjects and collected samples, oversaw the clinical management of patients, edited the original draft, supervised the project, and secured funding.

## FUNDING INFORMATION

This work was supported by the German Research Foundation (KFO296: AR232/25‐2, DI2103/2‐2) to P.A. and A.D., the Authority for Science, Research and Equality, Hanseatic City of Hamburg (State Research Funding, LFF‐FV73, Germany) to P.A., A.D., and T.Z., the Federal Ministry of Education and Research (BMBF, SRHOO junior research center for reproduction) to A.T., A.D., and P.A., and the Next Generation Partnership program and German Center for Child and Adolescent Health (funding code 01GL2404A) to P.A. and A.D. T.Z. is supported by the German Research Foundation, the EU Horizon 2020 program, the EU ERANet and ERAPreMed Programs, the German Center for Cardiovascular Research (DZHK e.V., 81Z0710102) and the German Ministry of Education and Research. T.Z. is listed as co‐founder and shareholder of the ART‐EMIS Hamburg GmbH, which holds an international patent application on the use of a computing device to estimate the probability of myocardial infarction (International Publication Numbers WO2022043229A1 and TW202219980A).

## CONFLICT OF INTEREST STATEMENT

All authors declare that they have no conflict of interest.

## ETHICS STATEMENT

The study protocol was approved by the Ethics committee of the Hamburg State Board of Physicians (2011 PV3694) on November 25, 2011 and conducted according to the principles of the Declaration of Helsinki. Written informed consent was obtained from all participants.

## Supporting information


Table S1.

Table S2.

**Figure S1.** Birthweight in relation to hs‐CRP tertiles in the individual trimesters. Scatterplot showing mean (thick horizontal line) with 95 % CI. First trimester: CRP 1st tertile 0.17 ‐ 2.49; 2nd tertile 2.49 ‐ 5.39; 3rd tertile 5.39 ‐ 62.1. Second trimester: CRP 1st tertile 0.21 ‐ 2.65; 2nd tertile 2.65 ‐ 5.22; 3rd tertile 5.22 – 127. 1st tertile 0.24 ‐ 2.24; 2nd tertile 2.24 ‐ 4.72;3rd tertile 4.72 ‐ 44.7.
