# Supplementary material for: High‐sensitivity CRP is elevated in pregnant women with overweight and obesity and modulated by gestational weight gain
Source: Acta Obstet Gynecol Scand. 2025 May 7;104(7):1339–46. doi: 10.1111/aogs.15135 (PMC12144584; doi:10.1111/aogs.15135)
Supplement: Supplementary file 1 — Table S1. [file AOGS-104-1339-s003.docx]

**Table S1. Metabolic biomarkers of the PRINCE cohort in the first trimester (n=582)**

|  | **First trimester median (IQ)** | **Laboratory reference range in a mixed population** |
| --- | --- | --- |
| **hsCRP (mg/L)** | 3.7 (1.9-7.0) | < 5 |
| **LDL (mg/dL)** | 102 (88-124) | 120-246 |
| **HDL (mg/dL)** | 70 (63-79) | > 60 |
| **Lp(a) (mg/dL)** | 9 (4-21) | < 30 |
| **Triglycerides (mg/dL)** | 97 (79-123) | < 150 |
| **hs-TnI (pg/mL)** | 0.9 (0.6-1.4) | < 14 |
| **Insulin (pmol/L)** | 50 (32-106) | 36-150 |
| **C-Peptid (ng/mL)** | 1.7 (1.1-2.8) | 0.8-4.2 |
| **Glucose (mg/dL)** | 75 (68-83) | 60-99 |
| **Apo A (g/L)** | 2.0 (1.8-2.2) | 1.1-2.3 |
| **Apo B (g/L)** | 0.9 (0.8-1.0) | 0.6-1.2 |
| **NT-proBNP (pg/mL)** | 65 (40-97) | < 125 |

All metabolic parameters (continuous variables), the quartiles are shown in the format: median (25th percentile, 75th percentile).
