# Supplementary material for: High‐sensitivity CRP is elevated in pregnant women with overweight and obesity and modulated by gestational weight gain
Source: Acta Obstet Gynecol Scand. 2025 May 7;104(7):1339–46. doi: 10.1111/aogs.15135 (PMC12144584; doi:10.1111/aogs.15135)
Supplement: Supplementary file 2 — Table S2. [file AOGS-104-1339-s002.docx]

**Table S2. Linear regression model for CRP and maternal age**

|  | **Beta (95% CI)** | **Beta per SD (95% CI)** | ***p*-value** |
| --- | --- | --- | --- |
| Overweight | 0.60 (0.39 - 0.81) | 0.25 (0.16 - 0.34) | <0.001 |
| Obese | 1.15 (0.86 - 1.43) | 0.35 (0.27 - 0.44) | <0.001 |
| Age | -0.02 (-0.04 - 0.01) | -0.06 (-0.15 - 0.03) | 0.16 |
| N | 421 |  |  |

Linear regression models were fitted and adjusted for age in order to evaluate the association between BMI groups and log-transformed CRP. A beta for the normal weight group is not included in the table as it is the reference group for overweight and obese. A statistically significant association between maternal age and elevated CRP values could be excluded using the linear regression model.
