# Supplementary figures and images for: High‐sensitivity CRP is elevated in pregnant women with overweight and obesity and modulated by gestational weight gain
Source: Acta Obstet Gynecol Scand. 2025 May 7;104(7):1339–46. doi: 10.1111/aogs.15135 (PMC12144584; doi:10.1111/aogs.15135)

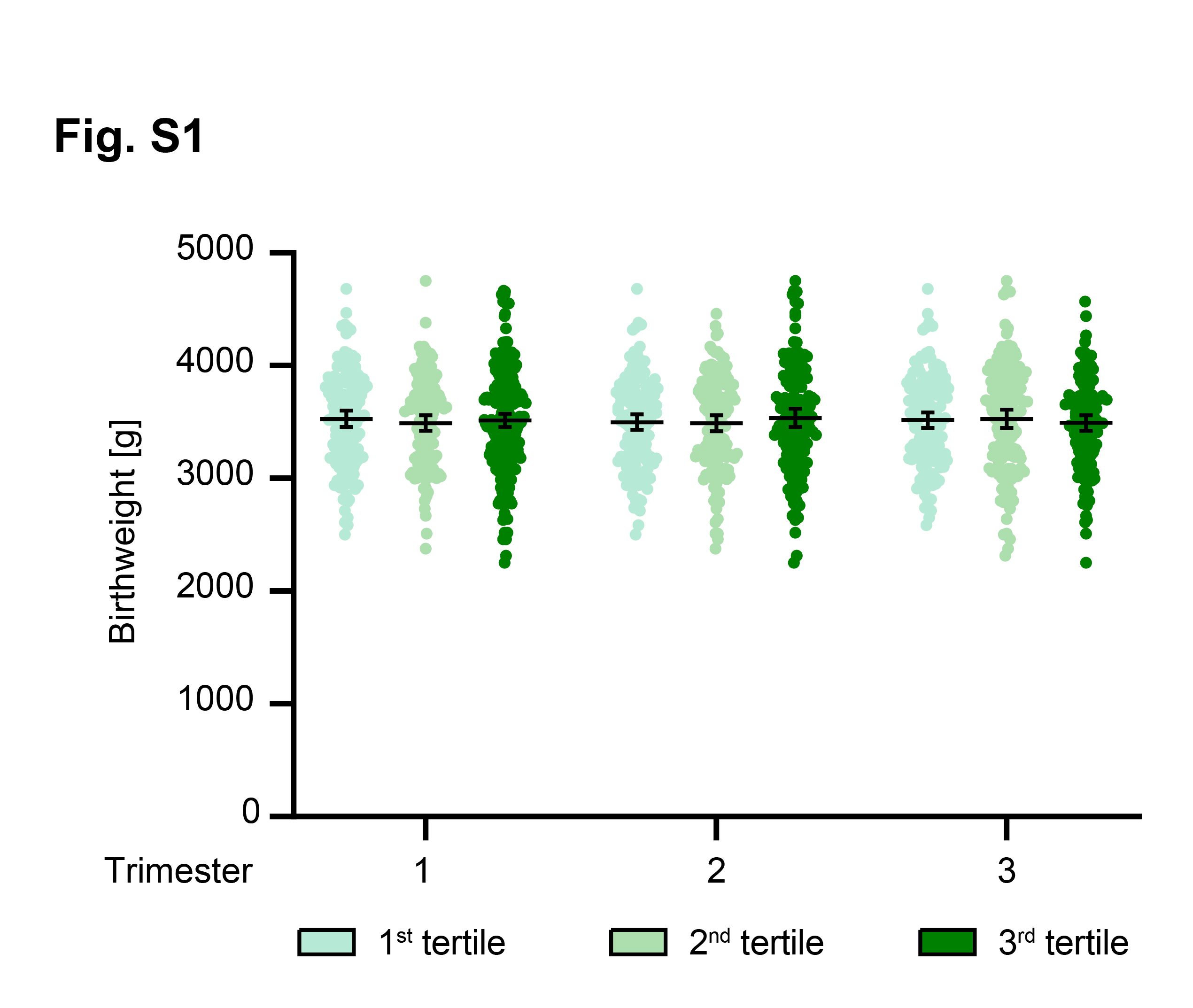

Supplement: Supplementary file 3 — Figure S1. Birthweight in relation to hs‐CRP tertiles in the individual trimesters. Scatterplot showing mean (thick horizontal line) with 95 % CI. First trimester: CRP 1st tertile 0.17 ‐ 2.49; 2nd tertile 2.49 ‐ 5.39; 3rd tertile 5.39 ‐ 62.1. Second trimester: CRP 1st tertile 0.21 ‐ 2.65; 2nd tertile 2.65 ‐ 5.22; 3rd tertile 5.22 – 127. 1st tertile 0.24 ‐ 2.24; 2nd tertile 2.24 ‐ 4.72;3rd tertile 4.72 ‐ 44.7. [file AOGS-104-1339-s001.png]
